# Supplementary material for: Chimpanzees adapt their exploration to key properties of the environment
Source: Nat Commun. 2025 Feb 20;16:1807. doi: 10.1038/s41467-025-57022-2 (PMC11842718; doi:10.1038/s41467-025-57022-2)
Supplement: Supplementary file 1 — Supplementary Information [file 41467_2025_57022_MOESM1_ESM.pdf]

Supplementary Information

for

Adaptive exploration in chimpanzees

Supplementary Methods

Proof of Concept Study

Prior to the actual experiment, we investigated whether chimpanzees are sensitive to environmental variance (Lejarraga et al., 2012) using a modified version of the information-seeking paradigm (Call & Carpenter, 2001; see also Beran & Smith, 2011; Bohn et al., 2017; Call, 2010; Call & Carpenter, 2001; Krachun & Call, 2009; Marsh & MacDonald, 2012; Rosati & Santos, 2016). In this paradigm, the experimenter hides a reward in one of two horizontal opaque tubes. Chimpanzees then choose one of the tubes. Critically, chimpanzees can watch the reward being hidden in some trials but not in others. In all trials, they can peek into the tubes before making a decision. The hypothesis is that if they are aware of their knowledge state (i.e., having or not having seen the reward being placed into the tube), they should search for information only in trials where they did not see the reward being hidden. The apes and children in the original study behaved as predicted, exploring significantly more often when they had not seen the reward being hidden. Broadening the questions addressed—from if and when primates seek information to what kind of information they seek, where it is acquired, and how adaptively the information source is used—offers an opportunity to expand the paradigm in terms of the complexity of tasks and the cognitive capacities involved (Marsh, 2019). In this proof of concept study, we tested whether chimpanzees explore more in uncertain conditions (with outcome variance) than in safe conditions (without outcome variance).

Methods

Participants. We tested eight semi-free-ranging chimpanzees from Sweetwaters Chimpanzee Sanctuary in Kenya (three females; age: *M* = 22.38 years, range = 16–35 years; for individual characteristics, see SourceData S01).

Animal welfare. The research was noninvasive and carried out in accordance with the guidelines of the Pan African Sanctuary Alliance and the regulations of Sweetwaters Chimpanzee Sanctuary, Ol Pejeta Conservancy, in Kenya. The full procedure of the study was approved by the local ethics committee at the sanctuaries (board members and veterinarian), Kenya Wildlife Service, and the Kenyan National Council for Science and Technology. Chimpanzees at the Sanctuary have access to large tracts of outdoor enclosures, including trees, bushes, and climbing structures, and live in large, mixed-sex social groups. They are fed a combination of fruits, vegetables, and other species-appropriate foods three times daily. All individuals stay in indoor enclosures overnight. Chimpanzees were tested in familiar rooms and never deprived of food or water for any reason. All testing was strictly voluntary. A chimpanzee could stop participating at any time by, for instance, heading to the door or not making a choice. All chimpanzees were highly motivated to participate.

Materials. The set-up consisted of two opaque buckets (height: 25.5 cm, diameter of opening: 30 cm), each with a lid, standing 200 cm apart from each other. A rope was attached to each lid and bucket.

Design. In a within-subjects design, chimpanzees were tested in a safe and an uncertain condition. Each condition comprised 24 trials (blocked) presented across four sessions. The order of conditions was counterbalanced across chimpanzees. The safe and uncertain option were not presented simultaneously; no choice was required between them. In both conditions, chimpanzees were presented with two buckets (options). In the safe condition, both buckets were baited with half an apple (i.e., both options were safe and none included outcome variance). In the uncertain condition, one bucket was baited with half an apple and one bucket was empty (outcome variance). The location of the food was counterbalanced across trials. Note that uncertainty here does not arise from choosing between a safe and an uncertain option (e.g., a choice between $3 for certain and $32 with a probability of 10% and $0 otherwise) but from not knowing which of the two options

holds the reward. Thus, outcome variance in this context does not refer to an option (a bucket) but rather to the whole trial (i.e., choosing one of the two buckets). In both conditions, the possible reward was the same: half an apple.

Test Phase. At the beginning of each trial, the experimenter showed the chimpanzee the contents of both buckets. In the safe condition, both buckets contained half an apple. In the uncertain condition, one bucket contained half an apple and the other bucket was empty. The experimenter then turned around, shuffled the buckets, and closed them with a lid. They then turned back to face the chimpanzee, placed both buckets in front of the chimpanzee, and made the lid ropes available. The chimpanzee had 20 seconds to explore the content of the buckets by pulling away the lids and standing up to peek into the buckets (eye line had to reach 1 m). After this exploration phase, the experimenter pulled the lids back onto the buckets and made the choice ropes available. The chimpanzee chose a bucket by pulling the respective choice rope. They received the reward if they chose the baited bucket and were shown the contents of the bucket they had not chosen. If no choice was made within 30 seconds, the trial was repeated. If a chimpanzee refused to make two choices in a row, the session was ended and the remaining trials were run the next day.

Coding and Reliability. To analyze whether chimpanzees explored more in the uncertain condition than in the safe condition, we coded whether chimpanzees pulled away the lids and stood up to peek into each bucket. All trials were recorded with one camera and coded live as well as later from video. A research assistant who was unaware of the study design and research question independently coded 20% of all trials. Interrater agreement was excellent for exploration (Cohen's k = 0.94) and choice (Cohen's k = 1).

Statistical Analysis

We employed Bayesian estimation techniques. Specifically, we conducted regression analyses using Bayesian generalized linear models implemented in R (R Core Team, 2023) using the Stan software, with the brm function from the brms package (Bürkner, 2017). We specified weakly informative normal priors with mean 0 and standard deviation 2 on all population-level effects (Gelman, 2006). We assessed the convergence of posteriors through visual inspection and the

Gelman–Rubin diagnostic, Rhat, with a cut-off value of 1.01 (Gelman & Rubin, 1992). In general, we report the mean of the posterior distribution of the parameter and two-sided 95% equal-tailed credible intervals (CI) around each value. We further computed Bayes factors to test whether the parameter differed from zero, using the hypothesis() function in the brms package (Wagenmakers et al., 2019). For all data figures, we used the function conditional_effects to display the conditional effects of the predictors of the fitted models (Bürkner, 2017). We computed leave-one-out cross-validation (LOO) values for every model (Vehtari et al., 2017). The LOO value indicates a model's pointwise out-of-sample prediction accuracy; models with higher LOO values are preferred. For the model comparison, we also added LOO weights (weights add up to 1). Following McElreath (2016), we used these metrics to rank models.

Results

Effect of variance. To investigate chimpanzees' exploration of the buckets, we used binomial logistic regression models. We ran three different models, with the response variable being exploration of the buckets (no, yes). In the first model (m1.0), we included the control predictors sex (female, male; reference category: male), age (in years), and order of condition (control first, test first; reference category: test first) as fixed effects, subject ID as a random intercept, and condition and trial number within subject ID as random slopes (but not the correlation of random slopes and intercept) (model notation in R: `searched ~ subject_sex + z.subject_age + order_condition + (1 + condition + z.trial | subject)`). In the next model (m1.1), we added condition (safe, uncertain; reference category: uncertain) to the control predictors of the first model (model notation in R: `searched ~ condition + z.trial + subject_sex + z.subject_age + order_condition + (1 + condition + z.trial | subject)`). In the final model (m1.2), we added an interaction term between condition (safe, uncertain; reference category: uncertain) and trial number within condition to the control predictors of the first model (model notation in R: `searched ~ condition * z.trial + subject_sex + z.subject_age + order_condition + (1 + condition + z.trial | subject)`). The covariates age and trial number were z-transformed. We set the adapt_delta parameter to 0.99

to ensure convergence of the models. All models were fitted using the Bernoulli family, and we ran each model with 4 chains, each with 4000 iterations (Table S1).

Overall, chimpanzees explored at least one bucket in 45% of trials: in 32% of trials in the safe condition and 57% in the uncertain condition. Model comparisons showed that models including the predictors condition and trial made better predictions, with the model including the interaction between condition and trials showing the best performance (interaction (m1.2): weight = 0.58; main effects (m1.1): weight = 0.42; without trial and condition (m1.0): weight = 0.00). The model estimate for the interaction term was positive (b = 0.47 [−0.06, 1.01]), suggesting that, over trials, chimpanzees increasingly explored more in the uncertain than in the safe condition. Because the corresponding 95% CI included 0, however, the estimate for the interaction term was associated with some uncertainty. We further computed a Bayes factor to test whether the parameter was greater than zero, using the hypothesis() function in the brms package (Wagenmakers et al., 2019). The results provide strong evidence (Evid.Ratio = 22.39; Post.Prob = 0.96) that the interaction between condition and trial significantly affected the probability of exploration in the binomial logistic regression model. In the context of the interaction model, the estimate for condition was reliably positive (b = 1.35 [0.31, 2.28]), suggesting that chimpanzees were more likely to explore the buckets in the uncertain condition than in the safe condition. The estimate for trial was reliably negative (b = −0.71 [−1.35, −0.16]).

The Present Experiment

In the present experiment, we investigated whether and how environmental change (RQ1; see Ruggeri et al., 2019) and outcome variance (RQ2; see Lejarraga et al., 2012) affect chimpanzees' exploration; which strategies they implement in their explorative behavior (RQ3; see Hills & Hertwig, 2010); and how individual characteristics, specifically risk and uncertainty preferences, shape chimpanzees' explorative behavior (RQ4; Mehlhorn et al., 2015; van den Bos & Hertwig, 2017). Finally, we investigated whether chimpanzees' decision making differs between environments.

Methods

Materials. A safe and an uncertain option, each comprising four small black trays (14 cm × 22 cm), were presented 115 cm apart from each other. A rope was attached to the lid of each tray. In the safe option, each tray was baited with a quarter of an apple (without outcome variance). In the uncertain option, two trays were baited with half an apple (two quarter pieces) each and two trays were empty (with outcome variance). We used two quarter pieces instead of half an apple to control for the mere preference for a larger piece and to make it easier for the chimpanzees to understand that the amount was double that in the safe option. After the exploration phase, the experimenter removed the lid ropes and made two choice ropes (one for each option) available. The chimpanzee chose one of the options by pulling the respective rope and received access to one tray drawn randomly from that option (see Figure 1 and Supplementary Movie 1).

Design. In a within-subjects design, chimpanzees were tested in a stable and a changing environment condition (Figure 1). In the stable environment condition, the safe and uncertain options stayed on the same sides across all trials and the same trays in the uncertain option were baited with food. In the changing environment condition, the safe and uncertain options changed sides over trials and the baiting of the trays in the uncertain option changed (six possible patterns for baiting two of four trays were implemented). Each condition comprised 32 trials (blocked), presented across eight sessions. The order of conditions was counterbalanced across

chimpanzees. The side of the safe option and the baiting pattern of the uncertain option was counterbalanced between subjects and within subjects.

In the decisions-from-experience paradigm established in research on risky choice in humans, participants are confronted with two options, each containing a set of outcomes that occur with some probability. In the simplest version, participants face an uncertain (with outcome variance) and a safe (without outcome variance) option. They are not told anything about the properties of the options, but are encouraged to explore them until they feel confident enough to decide which is “better.” When deciding between options, participants rely on subjective estimates of the (expected) values based on their explorative experience; final choices remain probabilistic (risky) choices. Even if they engage in extensive exploration, irreducible uncertainty remains (e.g., have all possible states of the world been encountered?) due to stochastic factors in the environment (aleatory uncertainty). In the information-seeking paradigm, in contrast, uncertainty is due to lack of knowledge (epistemic uncertainty; see Hacking, 1975).

Familiarization Phase. Prior to the test sessions, each chimpanzee was individually familiarized with the set-up. Familiarization consisted of a food quantity test using the same rewards as in the actual test (i.e., apples) followed by three consecutive pretest steps with bananas as rewards. During all familiarization steps but the last, the trays were presented without lids. Chimpanzees that passed the familiarization phase participated in the test trials.

Food quantity test. Each chimpanzee completed a food quantity test involving two consecutive sessions of four trials each, where they chose between a quarter of an apple and half an apple (two quarter pieces). The criterion was that they chose the tray baited with half an apple in seven of eight trials. For each trial, the rewards were placed on two separate trays, each furnished with a rope. Chimpanzees selected a tray by pulling its rope, then received the selected food. The nonselected food was removed and placed in a food bucket. The location of the half apple (left or right of the chimpanzee) was randomized and evenly distributed over trials. Three chimpanzees did not pass the food quantity test and were thus excluded from participation in the following pretests and experiment.

Pretest. All chimpanzees were individually introduced to the experimental set-up in three consecutive pretest steps.

In the first pretest (four consecutive sessions of eight trials each), chimpanzees were familiarized with the random mechanism. They experienced that they would receive only one of the four trays after each choice. The criterion was that they pulled the rope. For each trial, only one option was present. Its location (left or right of the chimpanzee), as well as which of the four trays was drawn, was randomized and evenly distributed over the trials. In the first two sessions, chimpanzees were familiarized with the safe option, in which each of the four trays were baited with a quarter of a banana. Chimpanzees were thus rewarded for pulling the rope on every trial. In the third and fourth sessions, chimpanzees were familiarized with the uncertain option, in which two trays were baited with half a banana each and two trays were empty. Chimpanzees were thus rewarded for pulling the rope in half of the trials. The reward pattern was pseudorandomized, with a maximum of two rewarded/nonrewarded trials in a row. In the third session, chimpanzees were exposed to a stable uncertain option (i.e., the same food pattern was presented across all trials); in the fourth session, they were exposed to a changing uncertain option (i.e., different food patterns were presented across trials).

In the second pretest (two consecutive sessions of eight trials each), chimpanzees were familiarized with the choice mechanism. They experienced that they could pull only one choice rope per trial. As in the first pretest, they experienced that they would receive only one of the four trays after each choice. In this pretest, however, both options were present in each trial. The criterion was that the chimpanzee pulled one of the choice ropes. Once they started pulling one rope, an experimenter removed the other. As in the first pretest, which of the four trays was drawn was randomized and evenly distributed over the trials. In a counterbalanced order across subjects, chimpanzees first chose between either two safe options (all eight trays baited with a quarter of a banana each; every trial rewarded) or two uncertain options (two trays baited with half a banana each, two trays empty; half the trials rewarded). The reward pattern was pseudorandomized, with a maximum of two rewarded/nonrewarded trials in a row.

In the third pretest (one session of four trials), chimpanzees were familiarized with the exploration mechanism. This was the first time they experienced closed trays. For one minute, they had the opportunity to explore the trays by opening their lids. There was no criterion. Unlike in the real test, chimpanzees did not receive any of the food rewards they saw. In a counterbalanced order across subjects, chimpanzees were either first confronted with one safe option and one uncertain stable option (i.e., two trials with the same food pattern) or with one safe option and one uncertain changing option (i.e., two trials with different food patterns). The location of the options (left or right of the chimpanzee) was randomized and evenly distributed over the trials. We decided against an explicit criterion in the third pretest because we did not want to “train” the chimpanzees to explore the options. However, across the four trials, chimpanzees explored between 7 and 31 trays. Thus, all chimpanzees demonstrated an understanding that they could pull the lid ropes to explore the options before entering the test.

Test Phase. The experimental procedure is an adaptation of the experience-based sampling paradigm used with humans (see Wulff et al., 2018). Chimpanzees began with no knowledge of the payoff distributions and could learn about the possible outcomes and their frequencies by drawing random samples from each option. This exploration process was under the chimpanzees' own control: They could decide whether to explore, which option to explore, and when to switch between options.

During the exploration phase, only the eight lid ropes were available. Chimpanzees had one minute to explore the content of all trays by pulling away the corresponding lids. After the exploration phase, the experimenter removed the opened lids and all lid ropes. Two experimenters then simultaneously provided the chimpanzee with the two choice ropes. Chimpanzees chose one of the options by pulling the corresponding rope (the other rope was pulled away as soon as a chimpanzee had touched one of the ropes). One tray was then randomly drawn from the option chosen, and the chimpanzee obtained the corresponding reward (or no reward if the selected tray was empty). If no choice was made within 30 seconds, the trial was repeated. If a chimpanzee refused to make a choice twice in a row, the session was ended and the remaining trials were run the next day.

Counterbalancing. In addition to the order of condition, we counterbalanced the side of the safe option (left or right), the reward pattern of the uncertain option (whether the uncertain option was rewarded on the left or right side; each side was rewarded equally often), the food patterns within the uncertain option (six possible patterns for baiting two of four trays), and which tray was pseudorandomly drawn from a chosen option.

Quantification of uncertainty. To obtain an objective uncertainty measure, we pseudorandomized the side of the safe and uncertain option and the food patterns (Fig S1.) according to the condition (stable environment; changing environment). Across the 32 trials of the stable environment for each chimpanzee the safe option was either always right or always left and for the uncertain option one of the six possible food patterns was administered (Fig S1.). We thereupon constructed six different patterns. These were counterbalanced across chimpanzees. There was just one kind of trial for each chimpanzee in the stable environment. The Shannon entropy (Shannon, & Weaver, 1949) of trials for the stable environment was zero (Signorell et al., 2019). For example, during the 32 trials (8 sessions) of the stable environment for the chimpanzee Akela, the safe option was always on the left side and the uncertain option always on the right side. The uncertain option was baited with half an apple in the second and fourth tray (pattern5, Fig S1.) across all trials. Across the 32 trials of the changing environment for each chimpanzee the safe option was on the left side in half of the trials. The six possible food patterns (Fig S1.) were distributed across the 32 trials. Within each chimpanzee each food pattern was administered at least twice and at most 4 times on each side. There were thus 12 different kinds of trials for each chimpanzee in the changing environment. We thereupon constructed six different patterns. These were counterbalanced across chimpanzees. The Shannon entropy (Shannon, & Weaver, 1949) of trials for the changing environment ranged between 3.53 and 3.56 (Signorell et al., 2019). For example, during the 32 trials (8 sessions) of the changing environment for the chimpanzee Akela the pattern was as follows (right/left indicates the location of the safe option; pattern1–6 the possible food patterns within the uncertain option): <u>session 1</u>: right_pattern6, left_pattern4, right_pattern3, left_pattern3, <u>session 2</u>: right_pattern5, left_pattern4, left_pattern5, right_pattern4, <u>session 3</u>: left_pattern5, right_pattern4, left_pattern6, right_pattern5, <u>session 4</u>: left_pattern6, left_pattern5,

right_pattern5, right_pattern2, session5: right_pattern3, left_pattern1, left_pattern1, right_pattern2, session 6: left_pattern3, left_pattern1, right_pattern6, right_pattern2, session 7: right_pattern4, left_pattern2, right_pattern6, left_pattern2, session 8: left_pattern4, right_pattern1, right_pattern1, left_pattern6.

	**tray 1**	**tray 2**	**tray 3**	**tray 4**
**pattern1**	1	2	3	4
**pattern2**	1	2	3	4
**pattern3**	1	2	3	4
**pattern4**	1	2	3	4
**pattern5**	1	2	3	4
**pattern6**	1	2	3	4

Figure S1. Six possible food patterns within the uncertain option. The black color indicates which trays were baited with half an apple.

Coding and Reliability. To assess how chimpanzees explore their environment, we coded whether and which trays were opened and which option was chosen. All trials were recorded with one camera and coded live as well as later from video. A research assistant who was unaware of the study design and hypotheses independently coded 20% of all trials. Interrater agreement was excellent for exploration (Cohen's k = 0.98) and choice (Cohen's k = 0.99).

Statistical Analysis

We employed Bayesian estimation techniques. Specifically, we conducted regression analyses using Bayesian generalized linear models implemented in R (R Core Team, 2023) using the Stan software, with the brm function from the brms package (Bürkner, 2017). We specified weakly informative normal priors with mean 0 and standard deviation 2 on all population-level effects (Gelman, 2006). We assessed the convergence of posteriors through visual inspection and the Gelman–Rubin diagnostic, Rhat, with a cut-off value of 1.01 (Gelman & Rubin, 1992). In general, we report the mean of the posterior distribution of the parameter and two-sided 95% equal-tailed credible intervals (CI) around each value. We further computed Bayes factors to test whether the parameter differed from zero, using the hypothesis() function in the brms package (Wagenmakers

et al., 2019). For all data figures, we used the function conditional_effects to display the conditional effects of the predictors of the fitted models (Bürkner, 2017). We computed leave-one-out cross-validation (LOO) values for every model (Vehtari et al., 2017). The LOO value indicates a model's pointwise out-of-sample prediction accuracy; models with higher LOO values are preferred. For the model comparison, we also added LOO weights (weights add up to 1). Following McElreath (2016), we used these metrics to rank models.

RQ1. Effect of environmental change. To investigate whether chimpanzees explored more in changing than in stable environments, we used binomial logistic regression models to examine their exploration of the trays within a trial. We ran three different models, with the response variable being the number of "yes" responses (total number of trays opened), modeled as a proportion of total trays (eight trays) per trial. In the first model (m2.0), we included the control predictors sex (female, male; reference category: male), age (in years), and order of condition (changing first, stable first; reference category: stable first) as fixed effects, subject ID as a random intercept, and condition and trial number within subject ID as random slopes (but not the correlation of random slopes and intercept) (model notation in R: `searched_yes | trials(total_trials) ~ subject_sex + z.subject_age + condition_order + (1 + condition + z.trial | subject)`). In the next model (m2.1), we added an interaction term between condition (changing, stable; reference category: stable) and trial number within condition to the control predictors of the first model (model notation in R: `searched_yes | trials(total_trials) ~ condition * z.trial + subject_sex + z.subject_age + condition_order + (1 + condition + z.trial | subject)`). In the final model (m2.2), we included condition and trial number within condition as separate predictors, in addition to the control predictors of the first model (model notation in R: `searched_yes | trials(total_trials) ~ condition + z.trial + subject_sex + z.subject_age + condition_order + (1 + condition + z.trial | subject)`). The covariates age and trial number were z-transformed. We set the

adapt_delta parameter to 0.99 to ensure convergence of the models. All models were fitted using the binomial family, and we ran each model with 4 chains, each with 4000 iterations (Table S2).

Model comparisons indicated that the model including the interaction between condition and trials showed the best performance (interaction (m2.1): weight = 0.71; main effects (m2.2): weight = 0; without trial and condition (m2.0): weight = 0.29). The model estimate for the interaction term was negative ($b = -0.21$ [−0.33, −0.09]), suggesting that, on average over trials, chimpanzees explored fewer trays in the stable than in the changing condition (Figure 2A). We further computed a Bayes factor to test whether the parameter was less than zero, using the hypothesis() function in the brms package (Wagenmakers et al., 2019). The results provide strong evidence (Evid.Ratio = inf; Post.Prob = 1) for a significant negative interaction between condition and trial. In the main effects model excluding the interaction, there was no effect of stable condition ($b = -0.03$ [−0.75, 0.67]) or trial ($b = -0.10$ [−0.34, 0.13]). The results suggest that over trials, when deciding between a safe and an uncertain option, chimpanzees explored changing environments more than stable environments. There were marked interindividual differences in exploration effort (Figure 2B).

RQ2. Effect of outcome variance. Next, we analyzed whether chimpanzees were more likely to open trays in the uncertain option than in the safe option during exploration, conditioned on them actually experiencing outcome variance in the former. Overall, chimpanzees explored (i.e., opened at least one tray) in 91% of trials. Within these trials, they experienced variance in the uncertain option in 62% of trials. Following Lejarraga et al. (2012), we first examined, for each trial, whether a chimpanzee experienced more than one kind of outcome within the uncertain option. In this case, we classified them as having experienced variance during this trial. Separating trials with and without the experience of outcome variance, we then calculated the difference between the number of opened trays in the uncertain versus the safe option in each of the 32 trials for both conditions and separately for each chimpanzee. A positive difference indicates more exploration in the uncertain than in the safe option. We ran three different Gaussian regression models, with the response variable being difference in opened trays between the uncertain and the safe option. In the first model (m3.0), we included the predictors experienced variance in the uncertain option (yes, no; reference category: yes), condition (changing, stable; reference category: stable), trial number

within condition as fixed effects, subject ID as a random intercept, and condition and trial number within subject ID as random slopes (but not the correlation of random slopes and intercept) (model notation in R: `difference_uncertain_safe ~ experienced_variance_uncertain + condition + z.trial + (1 + condition + z.trial | subject)`). In the next model (m3.1), we included the interaction term between experienced variance in the uncertain option (yes, no; reference category: yes) and condition (changing, stable; reference category: stable), in addition to the predictors of the model (model notation in R: `difference_uncertain_safe ~ experienced_variance_uncertain * condition + z.trial + (1 + condition + z.trial | subject)`). In the final model (m3.2), we included the interaction term between experienced variance in the uncertain option (yes, no; reference category: yes) and trial number within condition, in addition to the predictors of the first model (model notation in R: `difference_uncertain_safe ~ experienced_variance_uncertain * z.trial + condition + (1 + condition + z.trial| subject)`). The covariates age and trial number were z-transformed. We set the adapt_delta parameter to 0.99 to ensure convergence of the models. All models were fitted using the Gaussian family, and we ran each model with 4 chains, each with 4000 iterations (Table S3).

As Figure 2C shows, averaged across all chimpanzees, those that experienced outcome variance opened more trays in the uncertain option than in the safe option. In contrast, those that did not experience the variance of the uncertain option allocated more exploration effort to the safe option than to the uncertain option. Model comparisons showed that the first model showed the best performance, outperforming the models including interactions (main effect (m3.0): weight = 0.70; with interaction of experienced variance in the uncertain option and condition (m3.1): weight = 0.30, with interaction of experienced variance in the uncertain option and trial (m3.2): weight = 0.0). In the main effect model, the estimate for experienced variance in the uncertain option (yes, no; reference category: yes) was positive (b = 3.34 [3.07, 3.61]), suggesting that chimpanzees were significantly more likely to open trays in the uncertain than in the safe option when they had experienced outcome variance than when they had not. We further computed a Bayes factor to test whether the parameter was greater than zero, using the hypothesis() function in the brms package

(Wagenmakers et al., 2019). The results provided compelling evidence that experienced variance in the uncertain option had a significant positive effect on the difference in exploration between the risky and safe option (Evid.Ratio = Inf; Post.Prob = 1).

These results are in line with earlier findings suggesting that humans explore riskier options more than safer options when they experience outcome variance—a reasonable response to experienced risk (outcome variance; see Lejarraga et al., 2012). In the decisions-from-experience paradigm, participants are initially unaware of the possible outcomes and their variance, and learn about them through exploration. The relationship between sample size and experienced variance is thus bidirectional: Outcome variance within the uncertain option may lead to more exploration, but more exploration also leads to a greater likelihood of experiencing outcome variance. The direction of the causal relationship between experienced outcome variance and exploration could thus be confounded. To overcome this confound, we applied the analysis proposed by Lejarraga et al. (2012).

RQ3. Exploration strategies and switching behavior. We further investigated chimpanzee exploration and switching behaviors. Across subjects, chimpanzees explored only one option in half of the trials (*Mdn* = 0.48). When exploring both options, they preferentially explored sequentially (*Mdn* = 0.35)—that is, they only switched once, rather than employing a piecewise sampling strategy with multiple switches (*Mdn* = 0). In 9% of trials, chimpanzees did not explore at all (see Figure 2D). Looking at the two conditions separately, we found that exploring only one option and sequential exploration were the preferred strategies in both environments. Yet whereas both strategies were used equally often in the stable environment (one option: *Mdn* = 0.42; sequential exploration: *Mdn* = 0.44), there was a clear preference for sticking to one option (*Mdn* = 0.57) rather than exploring sequentially (*Mdn* = 0.35) in the changing environment (see Figure 2E). When they did explore both options, chimpanzees generally switched only once between options, consistent with the idea that switching behavior incurs energy and memory costs (see Hills & Hertwig, 2010). We then looked at both environments separately and found that in the changing environment, there was a clear preference for exploring only one option, whereas in the stable environment, exploration of one option and sequential exploration were used equally often. This is in line with previous

findings suggesting that if expectations about an option are violated, explorative behavior will be directed toward that option (e.g., Stahl & Feigenson, 2015). Focusing on one rather than multiple options can thus enhance learning without incurring the additional energy and memory costs of tracking change in different payoff distributions.

Delving deeper into the other environmental cues that potentially drove chimpanzees' switching, we investigated whether chimpanzees were more likely to switch to the safe option after finding food in the uncertain option. We ran three different binomial logistic regression models, with the response variable being switched option (yes, no). In the first model (m4.0), we included the control predictors condition (changing, stable; reference category: stable), trial number within condition, sex (female, male; reference category: male), age (in years) as fixed effects, subject ID as a random intercept, and condition and trial number within subject ID as random slopes (but not the correlation of random slopes and intercept) (model notation in R: `switched_assortment ~ condition + z.trial + subject_sex + z.subject_age + (1 + condition + z.trial | subject)`). In the next model (m4.1), we added the predictor previous sample found food (no, yes; reference category: yes) to the control predictors of the first model (model notation in R: `switched_assortment ~ prev_sample_found + condition + z.trial + subject_sex + z.subject_age + (1 + condition + z.trial | subject)`). In the final model (m4.2), we included the interaction term between previous sample found food (no, yes; reference category: yes) and condition (changing, stable; reference category: stable), in addition to the control predictors of the first model (model notation in R: `switched_assortment ~ prev_sample_found * condition + z.trial + subject_sex + z.subject_age + (1 + condition + z.trial | subject)`). The covariates age and trial number were z-transformed. We set the adapt_delta parameter to 0.99 to ensure convergence of the models. All models were fitted using the Bernoulli family, and we ran each model with 4 chains, each with 4000 iterations (Table S4).

Overall, chimpanzees switched to the safe option in 17% of trials after finding food (they continued to search in the risky option in 83% of trials) and in 12% of trials after not finding food (they continued to search in the risky option in 88% of trials). Model comparisons showed that

models including the predictor previous sample found food showed the best performance, outperforming the model including the interaction of condition and previous sample found food (main effect (m4.1): weight = 0.48; without previous sample found food (m4.0): weight = 0.28, interaction (m4.2): weight = 0.24). In the main effect model, the estimate for previous sample found food (no, yes; reference category: yes) was positive (b = 0.36 [0.05, 0.67]), suggesting that chimpanzees were more likely to switch to the safe option after finding food in the uncertain option. We further computed a Bayes factor to test whether the parameter was greater than zero, using the hypothesis() function in the brms package (Wagenmakers et al., 2019). The results strongly support the claim that previous sample found food was greater than zero (Evid.Ratio = 100.27; Post.Prob = 0.99). This "win–shift" strategy is adaptive in environments where food sources are dispersed (rather than clustered) or require time to be replenished: Obtaining food in one location decreases the likelihood of finding more food there (Kamil, 1978; Olton & Schlosberg, 1978). Relatedly, in experiments on risky choice, both the experience of a loss and the experience of gain systematically influenced nonhuman primates' choice of whether to gamble again in a next trial. Rosati and Hare (2013) found that bonobos, but not chimpanzees, modulated their choices based on previous outcomes. Haux et al. (2021) found that the outcome of the previous risky choice had a weak influence on the decision in the following trial: Chimpanzees tended to choose the risky (compared to the safe) option more often when they had previously received nothing. This may suggest that chimpanzee exploration and choice is guided not just by a process of reinforcement learning (in which the value of each action is updated according to its outcome) but also by a belief-updating process, in which the present outcome informs expectations about what will happen next (Haux et al., 2021).

RQ4. Correlations with risk and uncertainty preferences. As the same chimpanzees participated in both studies, we were further able to investigate the relationship between mean exploration effort (number of opened trays) in the present study and the chimpanzee risk and uncertainty preferences reported by Haux et al. (2023). Here, we calculated Pearson correlations and found no statistically significant correlation $t(11) = -.34$, $p = .74$, $r = -.10$, 95% CI = [−.62,.48] between chimpanzees' mean exploration effort and the behavioral risk measure and no statistically

significant correlation $t(12) = -1.70$, $p = .12$, $r = -{-}.44$, 95% CI = [−.79, .12] between chimpanzees' mean exploration effort and the observational risk measure for general risk. Furthermore, we found no statistically significant correlation $t(11) = 1.13$, $p = .28$, $r = .32$, 95% CI = [−.28, .74] between mean exploration effort and the behavioral uncertainty measure.

Choice behavior. Finally, we examined how chimpanzees decided between the safe and uncertain options across the two conditions. In the changing environment, their choices were split fairly equally between the two options, but in the stable condition there was a clear preference for the uncertain option, which was chosen in 75% of trials. We further investigated whether chimpanzees chose the uncertain option if they had *only* encountered an empty tray when exploring this option. Overall, this occurred in only 5% of trials, upon which chimpanzees chose the uncertain option in 59% and the safe option in 41% of trials. This behavior is also consistent with a clear preference for the uncertain option, assuming that sampling an empty tray was taken as a cue signaling the presence of the uncertain option.

Table S1. Proof of concept model outputs

		Estimate	Est. error	Lower 95% CI	Upper 95% CI	Rhat	Bulk_ ESS	Tail_ ESS	ELPD_LOO	SE	Weight
**Model 1.0**	**Draws:** 4 chains, each with iter = 4000; warmup = 2000; thin = 1; total post-warmup draws = 8000								–197.601	10.405	0.00
	intercept	−1.76	1.11	−4.01	0.48	1.00	2763	3622			
	subject male	−0.37	1.36	−3.01	2.32	1.00	4840	5276			
	z.subject age	−0.31	0.59	−1.51	0.90	1.00	4113	4253			
	uncertain condition first	1.45	1.42	−1.47	4.14	1.00	3673	4827			
**Model 1.1**	**Draws:** 4 chains, each with iter = 4000; warmup = 2000; thin = 1; total post-warmup draws = 8000								−196.389	10.668	0.42
	intercept	−1.73	1.07	−3.95	0.38	1.00	5087	5142			
	uncertain condition	1.32	0.51	0.23	2.28	1.00	4465	4207			
	z.trial	−0.48	0.27	−1.08	0.03	1.00	4617	4506			
	subject male	−0.15	1.46	−2.99	2.80	1.00	5112	5652			
	z.subject age	−0.34	0.68	−1.69	1.09	1.00	4413	4715			
	uncertain condition first	1.35	1.48	−1.69	4.15	1.00	3948	4888			
**Model 1.2**	Draws: 4 chains, each with iter = 4000; warmup = 2000; thin = 1; total post-warmup draws = 8000								−196.150	10.839	0.58
	intercept	−1.73	1.10	−3.92	0.41	1.00	5146	5581			
	uncertain condition	1.35	0.51	0.31	2.28	1.00	4704	4447			
	z.trial	−0.71	0.30	−1.35	−0.16	1.00	5394	4939			
	subject male	−0.15	1.43	−2.93	2.74	1.00	5595	5431			
	z.subject age	−0.35	0.69	−1.68	1.04	1.00	4787	5245			
	uncertain condition first	1.31	1.49	−1.71	4.19	1.00	4533	5475			
	uncertain condition*z.trial	0.47	0.27	−0.06	1.01	1.00	10639	6015			

The table summarizes the mean (Estimate), standard deviation (Est.error), and 95% credible intervals (Lower 95% CI and Upper 95% CI) of the posterior distribution. It also includes convergence (Rhat), effective sample size (Bulk_ESS, Tail_ESS), model fit (ELPD_LOO, SE), and model comparison metrics (Weight).

Table S2. Experiment model outputs: Effect of condition

		Estimate	Est. error	Lower 95% CI	Upper 95% CI	Rhat	Bulk_ ESS	Tail_ ESS	ELPD_LOO	SE	Weight
**Model 2.0**	**Draws:** 4 chains, each with iter = 4000; warmup = 2000; thin = 1; total post-warmup draws = 8000								−1703.217	41.619	0.29
	intercept	0.18	0.44	−0.68	1.08	1.00	3310	3449			
	subject male	−0.13	0.52	−1.15	0.91	1.00	3620	4210			
	z.subject age	0.10	0.28	−0.45	0.66	1.00	3428	4258			
	stable condition first	0.36	0.49	−0.63	1.34	1.00	2897	4450			
**Model 2.1**	**Draws:** 4 chains, each with iter = 4000; warmup = 2000; thin = 1; total post-warmup draws = 8000								−1697.992	42.227	0.71
	intercept	0.01	0.50	−0.99	1.00	1.00	5026	5048			
	stable condition	−0.07	0.38	−0.83	0.68	1.00	3868	4687			
	z.trial	−0.01	0.13	−0.26	0.24	1.00	5375	5369			
	subject male	−0.08	0.53	−1.16	0.97	1.00	5244	4789			
	z.subject age	0.11	0.27	−0.43	0.66	1.00	5137	4968			
	stable condition first	0.32	0.50	−0.68	1.33	1.00	5158	5046			
	stable condition*z.trial	−0.21	0.06	−0.33	−0.09	1.00	11087	5402			
**Model 2.2**	Draws: 4 chains, each with iter = 4000; warmup = 2000; thin = 1; total post-warmup draws = 8000								−1703.73	41.676	0.00
	intercept	0.02	0.48	−0.94	0.98	1.00	4416	4398			
	stable condition	−0.03	0.36	−0.75	0.67	1.00	3227	4336			
	z.trial	−0.10	0.12	−0.34	0.13	1.00	5048	5585			
	subject male	−0.14	0.53	−1.22	0.86	1.00	4938	4898			
	z.subject age	0.10	0.27	−0.45	0.65	1.00	4580	4703			
	stable condition first	0.35	0.50	−0.64	1.35	1.00	4519	5069			

The table summarizes the mean (Estimate), standard deviation (Est.error), and 95% credible intervals (Lower 95% CI and Upper 95% CI) of the posterior distribution. It also includes convergence (Rhat), effective sample size (Bulk_ESS, Tail_ESS), model fit (ELPD_LOO, SE), and model comparison metrics (Weight).

Table S3. Experiment model outputs: Outcome variance

		Estimate	Est. error	Lower 95% CI	Upper 95% CI	Rhat	Bulk_ ESS	Tail_ ESS	ELPD_LOO	SE	Weight
**Model 3.0**	**Draws:** 4 chains, each with iter = 4000; warmup = 2000; thin = 1; total post-warmup draws = 8000								−1486.571	21.455	0.70
	intercept	−1.83	0.22	−2.28	−1.37	1.00	1826	3669			
	experienced variance	3.34	0.14	3.07	3.61	1.00	9427	5960			
	stable condition	0.16	0.23	−0.30	0.62	1.00	3454	4533			
	z.trial	0.19	0.11	−0.01	0.41	1.00	3560	4031			
**Model 3.1**	**Draws:** 4 chains, each with iter = 4000; warmup = 2000; thin = 1; total post-warmup draws = 8000								−1486.902	21.720	0.30
	intercept	−1.91	0.24	−2.40	−1.45	1.00	3091	4771			
	experienced variance	3.47	0.18	3.13	3.82	1.00	7541	6282			
	stable condition	0.35	0.29	−0.24	0.92	1.00	4172	4862			
	z.trial	0.20	0.11	−0.02	0.41	1.00	4491	5304			
	experienced variance* stable condition	−0.32	0.27	−0.84	0.22	1.00	6124	5785			
**Model 3.2**	**Draws:** 4 chains, each with iter = 4000; warmup = 2000; thin = 1; total post-warmup draws = 8000								−1487.784	21.478	0.00
	intercept	−1.83	0.23	−2.28	−1.39	1.00	2680	4398			
	experienced variance	3.35	0.14	3.08	3.62	1.00	11635	6003			
	z.trial	0.17	0.14	−0.10	0.43	1.00	4525	4702			
	stable condition	0.15	0.24	−0.31	0.63	1.00	3740	4465			
	experienced variance* z.trial	0.04	0.13	−0.23	0.30	1.00	8504	6138			

The table summarizes the mean (Estimate), standard deviation (Est.error), and 95% credible intervals (Lower 95% CI and Upper 95% CI) of the posterior distribution. It also includes convergence (Rhat), effective sample size (Bulk_ESS, Tail_ESS), model fit (ELPD_LOO, SE), and model comparison metrics (Weight).

Table S4. Experiment model outputs: Effect of previous sample

		Estimate	Est. error	Lower 95% CI	Upper 95% CI	Rhat	Bulk_ESS	Tail_ESS	ELPD_LOO	SE	Weight
**Model 4.0**	**Draws:** 4 chains, each with iter = 4000; warmup = 2000; thin = 1; total post-warmup draws = 8000								−568.040	23.455	0.28
	intercept	−1.85	0.31	−2.48	−1.24	1.00	3296	4219			
	stable condition	−0.45	0.35	−1.21	0.19	1.00	3640	4087			
	z.trial	−0.04	0.09	−0.22	0.13	1.00	9112	5443			
	subject male	0.16	0.47	−0.81	1.10	1.00	3141	3568			
	z.subject age	−0.05	0.24	−0.53	0.43	1.00	2887	4249			
**Model 4.1**	**Draws:** 4 chains, each with iter = 4000; warmup = 2000; thin = 1; total post-warmup draws = 8000								−566.794	23.570	0.48
	intercept	−2.04	0.33	−2.69	−1.40	1.00	4078	4373			
	previous sample found food	0.36	0.16	0.05	0.67	1.00	13001	5933			
	stable condition	−0.44	0.35	−1.21	0.19	1.00	4197	4442			
	z.trial	−0.04	0.09	−0.21	0.14	1.00	10374	6443			
	subject male	0.15	0.47	−0.83	1.07	1.00	3140	4381			
	z.subject age	−0.05	0.24	−0.53	0.45	1.00	3311	4445			
**Model 4.2**	Draws: 4 chains, each with iter = 4000; warmup = 2000; thin = 1; total post-warmup draws = 8000								−567.084	23.597	0.24
	intercept	−1.96	0.33	−2.62	−1.32	1.00	4040	4613			
	previous sample found food	0.21	0.21	−0.20	0.64	1.00	6754	5697			
	stable condition	−0.62	0.39	−1.45	0.13	1.00	3402	3909			
	z.trial	−0.05	0.09	−0.22	0.13	1.00	6260	4884			
	subject male	0.17	0.46	−0.75	1.10	1.00	3216	4129			
	z.subject age	−0.05	0.25	−0.54	0.45	1.00	2571	3456			
	previous sample found food* stable condition	0.32	0.31	−0.30	0.91	1.00	6059	5745			

The table summarizes the mean (Estimate), standard deviation (Est.error), and 95% credible intervals (Lower 95% CI and Upper 95% CI) of the posterior distribution. It also includes convergence (Rhat), effective sample size (Bulk_ESS, Tail_ESS), model fit (ELPD_LOO, SE), and model comparison metrics (Weight).

Data availability. All data associated with this manuscript are available on GitHub: https://doi.org/10.5281/zenodo.13907943

Code availability. R scripts associated with this manuscript are available on GitHub. Exact computational reproducibility might only be achievable by using the model fit objects: https://doi.org/10.5281/zenodo.13907943

References for Supporting Information

Beran, M. J., & Smith, J. D. (2011). Information seeking by rhesus monkeys (*Macaca mulatta*) and capuchin monkeys (*Cebus apella*). *Cognition, 120*(1), 90–105. https://doi.org/10.1016/j.cognition.2011.02.016

Bohn, M., Allritz, M., Call, J., & Völter, C. J. (2017). Information seeking about tool properties in great apes. *Scientific Reports*, *7*(1), Article 10923. https://doi.org/10.1038/s41598-017-11400-z

Bürkner, P.-C. (2017). Advanced Bayesian multilevel modeling with the R package brms. *ArXiv*. https://arxiv.org/abs/1705.11123

Call, J. (2010). Do apes know that they could be wrong? *Animal Cognition, 13*(5), 689–700. https://doi.org/10.1007/s10071-010-0317-x

Call, J., & Carpenter, M. (2001). Do apes and children know what they have seen? *Animal Cognition, 3*(4), 207–220. https://doi.org/10.1007/s100710100078

Gelman, A. (2006). Prior distributions for variance parameters in hierarchical models (comment on article by Browne and Draper). *Bayesian Analysis*, *1*(3), 515–534. https://doi.org/10.1214/06-BA117A

Gelman, A., & Rubin, D. B. (1992). Inference from iterative simulation using multiple sequences. *Statistical Science*, *7*(4), 457–472. https://doi.org/10.1214/06-BA117A

Hacking, I. (1975). *Emergence of probability: A philosophical study of early ideas about probability, induction, and statistical inference.* Cambridge University Press.

Haux, L. M., Engelmann, J. M., Herrmann, E., & Hertwig, R. (2021). How chimpanzees decide in the face of social and nonsocial uncertainty. *Animal Behaviour*, *173*, 177–189. https://doi.org/10.1016/j.anbehav.2021.01.015

Haux, L. M., Engelmann, J. M., Arslan, R. C., Hertwig, R., & Herrmann, E. (2023). Chimpanzee and human risk preferences show key similarities. *Psychological Science*, *34*(3), 358–369. https://doi.org/10.1177/09567976221140326

Hills, T. T., & Hertwig, R. (2010). Information search in decisions from experience: Do our patterns of sampling foreshadow our decisions? *Psychological Science*, *21*(12), 1787–1792. https://doi.org/10.1177/0956797610387443

Kamil, A. C. (1978). Systematic foraging by a nectar-feeding bird, the Amakihi (*Loxops virens*). *Journal of Comparative and Physiological Psychology*, *92*(3), 388–396. https://doi.org/10.1037/h0077479

Krachun, C., & Call, J. (2009). Chimpanzees (*Pan troglodytes*) know what can be seen from where. *Animal Cognition, 12*(2), 317–331. https://doi.org/10.1007/s10071-008-0192-x

Lejarraga, T., Hertwig, R., & Gonzalez, C. (2012). How choice ecology influences search in decisions from experience. *Cognition*, *124*(3), 334–342. https://doi.org/10.1016/j.cognition.2012.06.002

Marsh, H. L. (2019). The information-seeking paradigm: Moving beyond 'if and when' to 'what, where, and how.' *Animal Behavior and Cognition*, *6*(4), 329–334. https://doi.org/10.26451/abc.06.04.11.2019

Marsh, H. L., & MacDonald, S. E. (2012). Information seeking by orangutans: A generalized search strategy? *Animal Cognition, 15*(3), 293–304. https://doi.org/10.1007/s10071-011-0453-y

McElreath, R. (2016). *Statistical rethinking: A Bayesian course with examples in R and Stan*. CRC Press.

Mehlhorn, K., Newell, B. R., Todd, P. M., Lee, M. D., Morgan, K., Braithwaite, V. A., Hausmann, D., Fiedler, K., & Gonzalez, C. (2015). Unpacking the exploration–exploitation tradeoff: A synthesis of human and animal literatures. *Decision*, *2*(3), 191–215. https://doi.org/10.1037/dec0000033

Olton, D. S., & Schlosberg, P. (1978). Food-searching strategies in young rats: Win–shift predominates over win–stay. *Journal of Comparative and Physiological Psychology*, *92*(4), 609–618. https://doi.org/10.1037/h0077492

R Core Team. (2023). R: A language and environment for statistical computing [Computer software]. R Foundation for Statistical Computing.

Rosati, A. G., & Hare, B. (2013). Chimpanzees and bonobos exhibit emotional responses to decision outcomes. *PloS One, 8*(5), Article e63058. https://doi.org/10.1371/journal.pone.0063058

Rosati, A. G., & Santos, L. R. (2016). Spontaneous metacognition in rhesus monkeys. *Psychological Science, 27*(9), 1181–1191. https://doi.org/10.1177/0956797616653737

Ruggeri, A., Swaboda, N., Sim, Z. L., & Gopnik, A. (2019). Shake it baby, but only when needed: Preschoolers adapt their exploratory strategies to the information structure of the task. *Cognition*, *193*, Article 104013. https://doi.org/10.1016/j.cognition.2019.104013

Shannon, C.E., Weaver, W. (1949). *The Mathematical Theory of Communication*. Univ of Illinois Press.

Signorell, A., Aho, K., Alfons, A., Anderegg, N., Aragon, T., Arppe, A., ... & Borchers, H. W. (2019). DescTools: Tools for descriptive statistics. R package version 0.99, 28, 17.

Stahl, A. E., & Feigenson, L. (2015). Observing the unexpected enhances infants' learning and exploration. *Science*, *348*(6230), 91–94. https://doi.org/10.1126/science.aaa3799

van den Bos, W., & Hertwig, R. (2017). Adolescents display distinctive tolerance to ambiguity and to uncertainty during risky decision making. *Scientific Reports*, *7*(1), Article 40962. https://doi.org/10.1038/srep40962

Vehtari, A., Gelman, A., & Gabry, J. (2017). Practical Bayesian model evaluation using leave-one-out cross-validation and WAIC. *Statistics and Computing*, *27*(5), 1413–1432. https://doi.org/10.1007/s11222-016-9696-4

Wagenmakers, E., Lee, M. D., Rouder, J. N., & Morey, R. D. (2019). The principle of predictive irrelevance, or why intervals should not be used for model comparison featuring a point null hypothesis. *PsyArXiv*. https://doi.org/10.31234/osf.io/rqnu5

Wulff, D. U., Mergenthaler-Canseco, M., & Hertwig, R. (2018). A meta-analytic review of two modes of learning and the description–experience gap. *Psychological Bulletin*, *144*(2), 140–176. https://doi.org/10.1037/bul0000115
