# Supplementary material for: Chimpanzees adapt their exploration to key properties of the environment
Source: Nat Commun. 2025 Feb 20;16:1807. doi: 10.1038/s41467-025-57022-2 (PMC11842718; doi:10.1038/s41467-025-57022-2)
Supplement: Supplementary file 2 — Description of Additional Supplementary Files [file 41467_2025_57022_MOESM2_ESM.pdf]

Description of Additional Supplementary Files

Supplementary Movie 1 - Experimental set-up.
