# Supplementary material for: Chimpanzees adapt their exploration to key properties of the environment
Source: Nat Commun. 2025 Feb 20;16:1807. doi: 10.1038/s41467-025-57022-2 (PMC11842718; doi:10.1038/s41467-025-57022-2)
Supplement: Supplementary file 4 — Reporting Summary [file 41467_2025_57022_MOESM4_ESM.pdf]

nature portfolio

Corresponding author(s): Lou M. Haux

Last updated by author(s): Oct 11, 2024

Reporting Summary

Nature Portfolio wishes to improve the reproducibility of the work that we publish. This form provides structure for consistency and transparency in reporting. For further information on Nature Portfolio policies, see our Editorial Policies and the Editorial Policy Checklist.

Statistics

For all statistical analyses, confirm that the following items are present in the figure legend, table legend, main text, or Methods section.

n/a	Confirmed	
☐	☒	The exact sample size (*n*) for each experimental group/condition, given as a discrete number and unit of measurement
☐	☒	A statement on whether measurements were taken from distinct samples or whether the same sample was measured repeatedly
☐	☒	The statistical test(s) used AND whether they are one- or two-sided<br>*Only common tests should be described solely by name; describe more complex techniques in the Methods section.*
☐	☒	A description of all covariates tested
☐	☒	A description of any assumptions or corrections, such as tests of normality and adjustment for multiple comparisons
☐	☒	A full description of the statistical parameters including central tendency (e.g. means) or other basic estimates (e.g. regression coefficient) AND variation (e.g. standard deviation) or associated estimates of uncertainty (e.g. confidence intervals)
☒	☐	For null hypothesis testing, the test statistic (e.g. *F*, *t*, *r*) with confidence intervals, effect sizes, degrees of freedom and *P* value noted<br>*Give P values as exact values whenever suitable.*
☐	☒	For Bayesian analysis, information on the choice of priors and Markov chain Monte Carlo settings
☐	☒	For hierarchical and complex designs, identification of the appropriate level for tests and full reporting of outcomes
☐	☒	Estimates of effect sizes (e.g. Cohen's *d*, Pearson's *r*), indicating how they were calculated

Our web collection on statistics for biologists contains articles on many of the points above.

Software and code

Policy information about availability of computer code

Data collection	No software was used for data collection
Data analysis	R version 4.4.1 (2024-06-14)<br><br>output of sessionInfo()<br>[1] ggpubr_0.6.0 sjPlot_2.8.16 writexl_1.5.0 apaTables_2.0.8 ggeasy_0.1.4 cowplot_1.1.3 plyr_1.8.9<br>[8] ggridges_0.5.6 kableExtra_1.4.0 psych_2.4.6.26 here_1.0.1 brms_2.21.0 Rcpp_1.0.12 lubridate_1.9.3 [15] forcats_1.0.0 stringr_1.5.1 dplyr_1.1.4 purrr_1.0.2 readr_2.1.5 tidyr_1.3.1 tibble_3.2.1<br>[22] ggplot2_3.5.1 tidyverse_2.0.0

For manuscripts utilizing custom algorithms or software that are central to the research but not yet described in published literature, software must be made available to editors and reviewers. We strongly encourage code deposition in a community repository (e.g. GitHub). See the Nature Portfolio guidelines for submitting code & software for further information.

Data

Policy information about availability of data

All manuscripts must include a data availability statement. This statement should provide the following information, where applicable:

- Accession codes, unique identifiers, or web links for publicly available datasets
- A description of any restrictions on data availability
- For clinical datasets or third party data, please ensure that the statement adheres to our policy

Data availability. The Adaptive Exploration data generated in this study are provided in the Supplementary Information/Source Data file.
Code availability. The data and R scripts associated with this manuscript are available on GitHub: https://doi.org/10.5281/zenodo.13907943

Research involving human participants, their data, or biological material

Policy information about studies with human participants or human data. See also policy information about sex, gender (identity/presentation), and sexual orientation and race, ethnicity and racism.

Reporting on sex and gender	The study did not involve humans
Reporting on race, ethnicity, or other socially relevant groupings	The study did not involve humans
Population characteristics	The study did not involve humans
Recruitment	The study did not involve humans
Ethics oversight	The study did not involve humans

Note that full information on the approval of the study protocol must also be provided in the manuscript.

Field-specific reporting

Please select the one below that is the best fit for your research. If you are not sure, read the appropriate sections before making your selection.

☐ Life sciences ☒ Behavioural & social sciences ☐ Ecological, evolutionary & environmental sciences

For a reference copy of the document with all sections, see nature.com/documents/nr-reporting-summary-flat.pdf

Behavioural & social sciences study design

All studies must disclose on these points even when the disclosure is negative.

Study description	We investigated exploration under uncertainty in chimpanzees by harnessing and adapting the human decisions-from-experience paradigm. Chimpanzees (N = 15) were simultaneously confronted with an uncertain (outcome variance) and a safe option (no outcome variance) and tested in both stable and changing environments. The data are quantitative.
Research sample	We tested 15 semi-free-ranging chimpanzees from Sweetwaters Chimpanzee Sanctuary in Kenya (eight females; age: M = 22.83 years, range = 13–33 years; for individual characteristics, see SourceData_S01_adaptive_exploration_chimpanzees).
Sampling strategy	Chimpanzees who passed the familiarization phase participated in the test trials. Due to the COVID-19 pandemic, 5% of the planned trials could not be run (see SourceData_S01_adaptive_exploration_chimpanzees for details on missing trials).
Data collection	To assess how chimpanzees explore their environment, we coded whether and which trays were opened and which option was chosen. All trials were recorded with one camera and coded live as well as later from video. A research assistant who was unaware of the study design and hypotheses independently coded 20% of all trials. Interrater agreement was excellent for exploration (Cohen's k = 0.98) and choice (Cohen's k = 0.99).
Timing	All data for the experiment were collected at Sweetwaters Chimpanzee Sanctuary between January and March 2020.
Data exclusions	Due to the COVID-19 pandemic, 5% of the planned trials could not be run (see SourceData_S01_adaptive_exploration_chimpanzees for details on missing trials). We excluded the data of one chimpanzee, Niyonkuru, as he was tested in only one condition.
Non-participation	We excluded the data of one chimpanzee, Niyonkuru, as he was tested in only one condition.
Randomization	The order of conditions was counterbalanced across chimpanzees

Reporting for specific materials, systems and methods

We require information from authors about some types of materials, experimental systems and methods used in many studies. Here, indicate whether each material, system or method listed is relevant to your study. If you are not sure if a list item applies to your research, read the appropriate section before selecting a response.

Materials & experimental systems

n/a	Involved in the study
☒	☐ Antibodies
☒	☐ Eukaryotic cell lines
☒	☐ Palaeontology and archaeology
☐	☒ Animals and other organisms
☒	☐ Clinical data
☒	☐ Dual use research of concern
☒	☐ Plants

Methods

n/a	Involved in the study
☒	☐ ChIP-seq
☒	☐ Flow cytometry
☒	☐ MRI-based neuroimaging

Animals and other research organisms

Policy information about studies involving animals; ARRIVE guidelines recommended for reporting animal research, and Sex and Gender in Research

Laboratory animals	The study did not involve laboratory animals. We tested semi-free-ranging chimpanzees from Sweetwaters Chimpanzee Sanctuary in Kenya.
Wild animals	The study did not involve wild animals. We tested semi-free-ranging chimpanzees from Sweetwaters Chimpanzee Sanctuary in Kenya.
Reporting on sex	We tested 15 semi-free-ranging chimpanzees from Sweetwaters Chimpanzee Sanctuary in Kenya (eight females; age: M = 22.83 years, range = 13–33 years; for individual characteristics, see SourceData_S01_adaptive_exploration_chimpanzees).
Field-collected samples	The study did not involve samples from the field.
Ethics oversight	Chimpanzees have access to large tracts of outdoor enclosures, including trees, bushes, and climbing structures, and live in large, mixed-sex social groups. All individuals stay in indoor enclosures overnight. Chimpanzees are tested in familiar rooms and are never deprived of food or water for any reason. All testing was strictly voluntary. During testing, a chimpanzee could indicate its wish to stop participating at any time by, for instance, heading to the door or not making a choice. All chimpanzees were highly motivated to participate. Chimpanzees are fed a combination of fruits, vegetables, and other species-appropriate foods three times daily. The research was noninvasive and carried out in accordance with the guidelines of the Pan African Sanctuary Alliance and the regulations of Sweetwaters Chimpanzee Sanctuary, Ol Pejeta Conservancy, in Kenya. The full procedure of the study was approved by the local ethics committee at the sanctuaries (board members and veterinarian), Kenya Wildlife Service, and the Kenyan National Council for Science and Technology.

Note that full information on the approval of the study protocol must also be provided in the manuscript.

Plants

Seed stocks	The study did not involve plants
Novel plant genotypes	The study did not involve plants
Authentication	The study did not involve plants
